# Payments by Patients

**Published:** 1899-07-15

**Authors:** 


### PAYMENTS BY PATIENTS.

### AN INTERESTING WILL CASE.

A curious will case, which ended in the division of a large sum of money between the two hospitals in St. Helens, Lancashire, was decided last Friday in the Chancery Court, Liverpool. The action arose from a dispute in regard to the will of the late Miss Ann Jane Garton, whose estate was sworn under £99,000, and it was understood that the action affected the disposal of about £40,000. The trustees of the will petitioned to obtain a declaration by the Court as to who was entitled to a legacy of £1,000 and one half of the residue, bequeathed in the will to the St. Helens (Lancashire) Infirmary, the petitioners having ascertained that there was no institution of that name, and the representatives of the St. Helens Hospital and the Providence Free Hospital at St. Helens having both claimed the legacy. The testatrix, who was a daughter of a late medical man in St. Helens, had lived in Southport 50 years, and under her will made bequests to Roman Catholic as well as Protestant schools and charities, though she was herself a Protestant. The Providence Free Hospital at St. Helens is conducted by a Roman Catholic sisterhood. Now there were several interesting points in this case. The first was the claim raised by the next-of-kin that, as there was no institution bearing the name, "St. Helens Infirmary," the money in dispute should go to them. No difficulty, however, was raised by the Vice-Chancellor in accepting the words "hospital" and "infirmary" as meaning the same thing. The trouble was to determine which of the two hospitals was meant. In regard to this it was urged by counsel for the St. Helens Hospital that confusion between the terms hospital and infirmary might easily arise, especially in the case of a person who had lived so long in Southport, where the "hospital" is a place for convalescents and the general hospital is called the "infirmary," and that the mere substitution of the word "hospital" for the word "infirmary," as it appeared in the will, made the wording of the will without further addition precisely applicable to the St. Helens Hospital, whereas the name of the Providence Free Hospital at St. Helens was entirely unlike the description given in the will. Moreover, it was pointed out that while the St. Helens Hospital possessed property and a certain amount of endowment, all of which was properly secured to its use under a trust deed, the Providence Free Hospital, although it was locally managed by a responsible committee, really belonged to a Roman Catholic sisterhood in London, who could, if they chose, close it at any moment. At this point it seemed as if all was in favour of the St. Helens Hospital getting the money. But there was another side to the question. The St. Helens Hospital was shown to be chiefly a provident institution, maintained principally, or largely, at any rate, for the use of those who subscribed, on the provident principle, so much a week, other patients having to pay for their maintenance and also for their medical attendance, and only accidents and urgent cases being admitted free; while the Providence Free Hospital was conducted on the ordinary principles of an eleemosynary hospital, the patients paying nothing for what they received, and the medical men of the town giving their services gratuitously. Thus the Court had to decide whether the St. Helens Hospital, which had commenced as a cottage hospital, and had in its larger sphere maintained the old principle of the patients paying in part at least for their maintenance and their medical attendance, was to be considered as a charity such as the testatrix contemplated, and whether the Providence Free Hospital, notwithstanding its difference of name, did not more fully occupy the place and fulfil the functions of an infirmary or hospital, such as was contemplated by the testatrix, seeing that it was open free to all sorts of patients whether subscribers or not. The Vice-Chancellor suggested a compromise, but on this the counsel for the next-of-kin claimed that if the judge could not decide between the claims of the two institutions, the bequest should fail, and the money should come to the next-of-kin. On this a long legal argument ensued, much of it turning on the question whether or not there was shown in the will a general charitable intention. In regard to this the Vice-Chancellor pointed out that everything except £2,000 and all the residue went to charity. The legacy was, he said, either given to the St. Helens Hospital or else to the two institutions to divide between them. The next-of-kin were out of it, but he thought they ought to have their costs, as between solicitor and client. The question thus came back to that between the two hospitals, and on it being announced that they had come to an arrangement judgment was given in favour of the St. Helens Hospital on condition of £6,000 being given to the endowment fund of the Providence Free Hospital, the St. Helens Hospital to pay the costs. The details of the final arrangements will, however, have to be brought up again to receive the sanction of the Court, and in regard to this the Vice-Chancellor intimated that he should require that the money to be received by the Providence Free Hospital should in some form or another be settled by deed and held in trust for the permanent carrying on of a hospital at St. Helens; in other words, that it was for the hospital and not for the sisterhood. The chief point of interest in this case, so far as hospital management is concerned, is that the Court held that neither the payment by the patients of a certain sum per week while in the hospital, nor the partial restriction of the benefits of the hospital to the provident subscribers to its funds, nor the payment of the medical fees by the patients, placed the institution outside the definition of a charity, and that these conditions did not interfere with its receiving money under a will which was clearly made out with "a general charitable intention." This may be a matter of importance to some cottage hospitals.

